# VNTRseek—a computational tool to detect tandem repeat variants in high-throughput sequencing data

**DOI:** 10.1093/nar/gku642

**Published:** 2014-07-23

**Authors:** Yevgeniy Gelfand, Yozen Hernandez, Joshua Loving, Gary Benson

**Affiliations:** 1 Laboratory for Biocomputing and Informatics, Boston University, Boston, MA 02215, USA; 2 Graduate Program in Bioinformatics, Boston University, Boston, MA 02215, USA; 3 Department of Computer Science, Boston University, Boston, MA 02215, USA

## Abstract

DNA tandem repeats (TRs) are ubiquitous genomic features which consist of two or more adjacent copies of an underlying pattern sequence. The copies may be identical or approximate. Variable number of tandem repeats or VNTRs are polymorphic TR loci in which the number of pattern copies is variable. In this paper we describe VNTRseek, our software for discovery of *minisatellite* VNTRs (pattern size ≥ 7 nucleotides) using whole genome sequencing data. VNTRseek maps sequencing reads to a set of reference TRs and then identifies putative VNTRs based on a discrepancy between the copy number of a reference and its mapped reads. VNTRseek was used to analyze the Watson and Khoisan genomes (454 technology) and two 1000 Genomes family trios (Illumina). In the Watson genome, we identified 752 VNTRs with pattern sizes ranging from 7 to 84 nt. In the Khoisan genome, we identified 2572 VNTRs with pattern sizes ranging from 7 to 105 nt. In the trios, we identified between 2660 and 3822 VNTRs per individual and found nearly 100% consistency with Mendelian inheritance. VNTRseek is, to the best of our knowledge, the first software for genome-wide detection of minisatellite VNTRs. It is available at http://orca.bu.edu/vntrseek/.

## INTRODUCTION

DNA tandem repeats (TRs) are typically divided into two classes, *microsatellites* which have short pattern sizes, generally 1–6 nucleotides (nt), and *minisatellites* which have longer patterns. Tandem repeat variants, or VNTRs (variable number of tandem repeats), are loci in which the number of internal copies in the repeat varies in the population. When compared to a reference genome, VNTRs look like indels with one or more copies of the underlying pattern gained or lost.

VNTRs often find use as genomic markers because of their abundance and plasticity. For example, microsatellites can have mutation rates from one thousand to one-hundred thousand times higher than single nucleotide polymorphisms (1–3) and minisatellites with high heterozygosity rates have been confirmed in the human and other genomes (4).

### Phenotypic role of VNTRs

VNTRs are known to affect gene expression, disease states and chromatin structure. Several examples are described below.

#### Effect on transcriptional variability

In *Saccharomyces cerevisiae*, among the quarter of gene promoters containing TRs, those with VNTRs exhibit elevated expression divergence, a measure of the speed with which transcriptional activity evolves (5). Direct testing of expression in two yeast genes, *YHB1* and *MET3*, following imposed variation in TR array length revealed length dependent changes in gene expression. (A TR *array* is the entire repeat sequence containing the multiple adjacent copies.)

#### Association with disease

At least 17 diseases are the result of unstable trinucleotide TRs, including fragile-X mental retardation (6), Huntington's disease (7), myotonic dystrophy (8) and Friedreich's ataxia (9). Examples for minisatellite loci include two distinct VNTRs in the serotonin transporter gene (5-HTT, also SLC6A4) which have been linked to phenotype in a variety of populations: HTTLPR, a 20–23 bp TR in the promoter, which occurs as 14 copies and 16 copies, and STin2, a 17 bp TR in the second intron, which occurs as 9, 10 and 12 copies. HTTLPR variants have been associated with depression (10), bipolar disorder (11), symptoms of Alzheimer's disease (12) and may act through a decrease in transcriptional activity of the gene in patients with the shorter allele (13). STin2 has been associated with schizophrenia (14), depression (15), symptoms of Alzheimer's disease (12,16) and treatment outcome in alcohol dependent patients (17).

#### Allele silencing

Paramutation (18) is an interaction between alleles at the same locus in which one allele silences the other, typically through a change in chromatin state. The change is *heritable*, that is, the silenced allele remains inactive even after transmission to the next generation, where it can act as a silencer of an inherited active allele. In maize, a minisatellite VNTR is pivotal to paramutation at the *b1* locus, which encodes a transcription factor affecting plant pigmentation. Seven tandem copies of an 853 bp pattern are located 100 kb upstream of the gene and loss of copies lessens or extinguishes the silencing effect (19,20). Production of siRNAs with the TRs as templates, RNA interference, and cytosine methylation are all involved (20–23). Long TRs are similarly involved in paramutagenic silencing of the *FWA* flowering time regulator gene in *A. thaliana* (24–26).

### Variant detection software

VNTRs have received only limited study on a genome-wide scale due to a lack of specialized tools for their detection (27). Accurate mapping of VNTRs using high-throughput sequencing data is problematic for standard read mapping software, e.g. (28–30), because copy changes appear as moderate to large indels and mapping programs, which are optimized for speed, are designed to deal with limited or no indels. Indel discovery programs, e.g. (31–34), have difficulty mapping variants because of similarities and subtle differences in the adjacent copies. Both types of programs are generally annotation oblivious, i.e. they do not know that a read maps to a TR, are not designed to distinguish internal copy number differences from smaller changes, and do not visualize the variants in ways that can enhance understanding of repeat mutation dynamics.

Recently, several groups have developed targeted methods to detect polymorphic *microsatellite* TRs using genome-wide, high-throughput sequencing data (35–41). The Garner lab's method (35,40), uses a combination of BLAST (42), BWA (28) and custom scripts to align reads to a microsatellite reference set. lobSTR (38) uses its own mapping approach and applies a model to correct for variant allele length introduced by polymerase chain reaction (PCR) amplification. RepeatSeq (36,37) uses a combination of Novoalign or Bowtie2 (43), GATK IndelRealigner (44), and a custom Bayesian approach which incorporates prior knowledge of genotype calling errors. Most methods use the Tandem Repeats Finder (TRF) program (45) to identify TRs in the reads and to establish reference sets.

At the other end of the size spectrum, in the 1–10 kb range, an algorithm (46) has been been reported to detect *de novo* tandem duplications, i.e. the locus is not a repeat in the reference genome. Other programs exist to detect large, non-tandem copy number variants, e.g. (47–50).

Between the extremes of microsatellites and large structural duplications, the focus of the present work is discovery of *minisatellite* TRs with patterns sizes ≥7 nt and up to several hundred nt, as limited by read length. Our goal has been to develop specifically targeted, efficient VNTR detection software, which can provide essential information on VNTR occurrence and characteristics. Our program, *VNTRseek*, is, to the best of our knowledge, the first software for genome-wide detection of minisatellite VNTRs. In outline, it works as follows: (i) TRF is used to identify a reference set of TR loci and to identify TRs in the reads; (ii) the read TRs are mapped to the reference TRs based on similarity in the repeat consensus patterns, and the TR array profiles; (iii) mappings are confirmed based on comparison of the read and reference flanking sequences, adjacent upstream and downstream to the TR arrays; and (iv) TR genotypes are called based on the number of pattern copies in the mapped reads. In particular, a locus is called a VNTR if it has at least two mapped reads which exhibit a common copy number different from that in the reference.

## MATERIALS AND METHODS

### Reference genome

The TRF program (45) was run on the Human genome sequence hg19 (Build 37, February 2009) downloaded from the UCSC genome ftp download page. Only files of type chrxxx.fa.gz where xxx is one of [1,…,22, X, Y, M] were processed. TRF command line parameters used were 2 5 7 80 10 50 2000 (match weight, mismatch penalty, indel penalty, match probability, indel probability, minimum score, maximum period size).

The results were filtered in the Tandem Repeats Database (51) to remove (i) low-quality TRs with many indels and mismatches (average per column alignment score ≤ 1.3); (ii) TRs having significant overlap (>20% of total length overlapping) with common interspersed repeat elements including SINEs, LINEs, LTRs and DNA transposons identified by RepeatMasker (http://www.repeatmasker.org); (iii) redundant TRs reported for the same locus using the TRDB Redundancy Elimination tool, and (iv) microsatellite TRs (pattern size ≤ 6). For redundancy elimination, if TRs overlapped by more than 50% of their length, the repeat with the longer array was retained, or in the case of ties, the repeat with the shorter period was retained. The set after these filters contained 230,671 TRs. 365 TRs were subsequently removed to reduce the incidence of false positive VNTRs detected while mapping simulated reads (see Results). The final reference set contained 230,306 TRs (ref-TRs).

### Subject data

The Watson 454 data (52) consisted of 74,198,831 reads obtained from TraceDB (ftp.ncbi.nih.gov/pub/TraceDB/Personal_Genomics/Watson/), with an average length of 261 nucleotides (nt) and a nominal coverage of 6.26. The Khoisan 454 data (53) consisted of 83,331,227 reads obtained from NCBI's Short Read Archive (ftp-trace.ncbi.nlm.nih.gov/sra/sra-instant/reads/ByExp/sra/SRX/SRX015/SRX015665) with an average length of 565 nt and a nominal coverage of 15.69. SRA files were converted to FASTQ using the fastq-dump utility from NCBI's SRA Toolkit. Illumina 250 bp, PCR free, paired-end reads for two 1000 Genomes trios (mother-father-daughter), one a Utah family (NA12878, NA12891, NA12892) of European ancestry and the other a Nigerian Yoruban family (NA19238, NA19239, NA19240), with coverage ranging from 68-81 were obtained from the European Nucleotide Archive (www.ebi.ac.uk/ena/data/view/PRJNA196624 and www.ebi.ac.uk/ena/data/view/PRJEB4252). Each read from all data sets was processed with TRF using the same parameters used to process the reference genome as well as the flags -d -h -ngs. The results were filtered to retain only those reads containing a minisatellite repeat (pattern size ≥ 7) with at least 20 nt of flanking sequence on each side (read-TR). The final Watson data set contained 2,925,732 reads and 4,826,849 read-TRs. The final Khoisan data set contained 15,009,889 reads and 59,601,310 read-TRs. The final trios data sets contained 33,279,934 – 39,599,364 reads and 60,350,672 – 68,388,983 read-TRs.

### Profile representation

For subsequent alignment steps, each TR was represented by a *normalized profile*, *P*, obtained from a multiple alignment of the individual pattern copies (54),

\begin{eqnarray*} P= C_1C_2, \cdots C_k \mbox{ with } C_i = (n_A,n_C,n_G,n_T,n_{-}), \end{eqnarray*}

where *k* is the number of columns in the multiple alignment. *C*_*i*_ is a normalized vector of counts of nucleotides in column *i* where *n*_σ_ denotes the count for nucleotide σ and *n*_−_ the count for gaps (N's are ignored). Instead of actual counts, the counts in *C*_*i*_ always add to 10. Actual counts were first converted proportionately to counts with a sum of 10 and then mapped to the closest of the 1001 possible normalized vectors as measured by Euclidean distance. The normalized reverse complement profile was obtained in an identical way starting with a multiple alignment of the reverse complement TR sequence.

### Read mapping and VNTR calling

#### Alignments

Spaced-seed indexing (55,56) of the TR consensus patterns was used to determine candidate pairings of read-TRs to ref-TRs. Pairings were confirmed with three types of alignment: (i) longest common subsequence (LCS) comparison of consensus patterns; (ii) profile alignment of TR arrays; and (iii) edit-distance alignment of flanking sequences.

Consensus pattern LCS comparison was used as a quick pre-filter for the remaining alignments. A threshold LCS length of at least 85% of the shorter pattern was required and a maximum length difference of 10% was allowed between patterns. LCS was computed with a fast bit-parallel algorithm (57).

For profile alignment, Euclidean distance scoring between pairs of normalized count vectors was used as described in (58) and computed with a narrowband technique (59). To prevent vectors with high dash counts, due to deleted or inserted characters, from contributing significantly to the score, the distance score was converted to a weighted distance as follows:

\begin{eqnarray*} &&{\rm WD}(P_1,P_2) = \sum _i E_i(V,W)*{\rm Weight}_i \\ &&{\rm Weight}_i = \min (10,20 -({\rm gaps}(V)+{\rm gaps}(W))) \end{eqnarray*}

where *P*_1_, *P*_2_ are profiles, *E*_*i*_(*V*, *W*) is the Euclidean distance for matched count vectors *V* from *P*_1_ and *W* from *P*_2_ at alignment position *i*, Weight_*i*_ is the weight applied at position *i*, with a maximum of 10 and a minimum of zero, and gaps(*) is the number of gap characters in a vector out of 10. The weighted distance score was then converted to a pseudo-similarity score, WS, between 0 (worst) and 100 (best):

\begin{equation*} {\rm WS}(P_1,P_2) = 100-\left[{{{\rm WD}(P_1,P_2)}\over 255 * \sum _i {\rm Weight}_i}*100\right] \end{equation*}

where *P*_*i*_ is a profile, and 255 is the worst possible Euclidean distance weight. A WS threshold of at least 88 was required.

For flanking sequence alignment, unit-cost edit distance scoring was used with a required threshold of ≤10% errors relative to the combined lengths of the read-TR flanking sequences. Unit-cost edit-distance was computed with a fast bit-parallel algorithm (improved from (60) and available for download at http://lobstah.bu.edu/cgi-bin/BitPAl/getedit.py).

#### Mapping

Read-TR/ref-TR pairs passing the alignment filters were retained for mapping and were sorted into two lists, one by profile WS score and one by flank score. Within each list, (i) each read-TR picked its best scoring ref-TR (or all best scoring if ties occurred) and then (ii) each ref-TR picked its best read-TR if more than one occurred in the same read (or in the case of ties, the highest numbered read-TR). The lists were then merged by intersection so that a read-TR was mapped to a ref-TR only if that ref-TR yielded the best profile and flank score. In case of ties for a read-TR, the pair was removed from the output. Finally, reads were discarded if they mapped to three or more ref-TRs or to two ref-TRs that were too far apart for the read length.

#### PCR duplicate elimination

After mapping, all reads mapped to the same ref-TR were compared to eliminate PCR duplicates. Two reads were considered duplicates if their lengths differed by no more than two nucleotides and they had an LCS no shorter than the shorter read length minus 3.

#### Indistinguishable ref-TRs

Families of highly similar TRs are common in many genomes. Repeats within a family often share homology in their arrays and flanking sequences. When a read originates from one member of a TR family, other family members in the reference set typically score well in both profile and flanking sequence alignments and the mapping can be uncertain. To flag this situation, the ref-TR set was processed against itself through the alignment steps with thresholds as described above, except the flanking sequence alignment threshold required ≤10% errors for flanks of exactly 50 nt *on only one side*. Any ref-TR that passed the filters for a reference other than itself was classified as *indistinguishable*. All others were classified as *singletons*.

#### Allele Support and VNTR Calling

An allele containing *n* tandem copies was considered to have *support* if the ref-TR had at least two mapped reads with *n* copies. Ref-TRs with supported alleles were categorized as follows:

1. Single allele, same as reference—Not a VNTR.
2. Single allele, different from reference—An *inferred* VNTR assuming that the reference is correct, i.e. not an artifact.
3. Two alleles—An *observed* VNTR.

### Performance and validation

#### Simulated reads

Read sets were generated, with locations determined by a 64-bit Mersenne Twister pseudo random number generator (61) (www.math.sci.hiroshima-u.ac.jp/∼m-mat/MT/emt64.html) assuming a diploid genome with chromosome lengths and sequences matching the hg19 reference (www.ncbi.nlm.nih.gov/projects/genome/assembly/grc/human/data/?build=37). For 454 simulation, read sets consisted of 74,000,000 reads with lengths drawn randomly from a normal distribution as generated using an implementation of the Box Muller Polar transform method (www.taygeta.com/random/gaussian.html), assuming a mean length of 261 nt and a standard deviation of 27 nt. The resulting read sets closely matched the Watson data set in read number and read length. For Illumina simulation, read sets consisted of 193,000,000 reads with lengths uniformly set to 100 nt (in order to achieve the same coverage as the 454 reads). To introduce sequencing errors, a simulated read set was first produced and then the sequences were modified using a technology specific error model. For 454 reads, error rates were derived from empirical data on the 454 GS FLX sequencer, as described in (62). The error rates were homopolymer length-dependent, with indel error types (overcalls and undercalls) at much higher frequency than substitution errors, and with the probability of error increasing with homopolymer length. Error rates were applied on a per-homopolymer basis, with each homopolymer of length *n* ≥ 1 having a random chance of being over or undercalled, and homopolymers of length one having a random chance of undergoing a substitution. For Illumina reads, error rates were similarly derived from empirical data also described in (62). Substitution errors were position dependent, with the rate of substitution increasing with the base position. Indel errors were single-base and independent of base position.

#### Simulated VNTRs

A modified reference set was created for testing homozygous detection of VNTRs by changing the copy number of 1118 randomly selected ref-TRs (0.5% random selection frequency, similar to the frequency of Watson detected VNTRs) so that they had one or two pattern copies added or deleted. In each case, existing copies within a selected ref-TR were randomly selected for duplication or removal. This only affected the copy count, repeat array, and repeat profile in these references and did not affect the chromosomes from which the reads were drawn. For testing heterozygous detection, two sets of hg19 chromosomes were used. One set was unmodified, the other set was modified by changing the TR arrays to those in the 1118 modified ref-TRs. Simulated reads were drawn equally from the modified and unmodified chromosomes and mapped back to the unmodified reference set.

#### Calculation of expected number of spanned TR loci

A probability formula was developed to determine the expected number of TR-loci, of a given array size, that would be spanned by a read set with fixed read size. See Supplementary Material for details.

#### Simulation of expected number of spanned TR loci

The same technique as described for simulated 454 reads above was used in 50 trials generating 74,000,000 reads for each trial. These reads were not mapped. Instead their origins were compared to the locations of the ref-TRs. For each ref-TR in each trial, it was determined if at least one read was generated that spanned the TR. Reported was the average number of times each locus was spanned, with loci grouped by array length.

#### BLAST comparison of Watson data

Two BLAST databases were built from the Watson reads, one containing all the reads, the other containing only reads found by TRF to contain TRs with pattern size ≥7 and with ≥20 nt of flanking sequence on each side of the TR array. Queries were performed against the databases using ref-TRs that had no reads assigned to them after the profile and flank alignment steps. Other ref-TRs that had reads assigned, but lost those reads to another reference during mapping or due to ties, were excluded. Query sequences consisted of the ref-TR array and 20 nt of flanking sequence on each side. BLAST default parameters were used. A hit was counted if the high-scoring segment pair (HSP) contained at least 98% of the query sequence.

#### Comparison to Watson indels from dbSNP

Watson indel data were obtained from dbSNP and the dbSNP Batch Query page (ftp.ncbi.nih.gov/snp/organisms/human_9606/viewBatch/snpBatch_BCMHGSC_JDW_60451.gz). Locations of the indels were compared to locations of the ref-TRs to determine overlaps. Alignment between indel sequences and ref-TR consensus patterns were used to determine if the indels were consistent with single or multiple pattern gain or loss.

## RESULTS

### Performance

We evaluated the performance of VNTRseek in five ways: (i) simulation studies to determine nominal accuracy for read mapping and VNTR calling, (ii) comparison of the expected number of mapped ref-TRs to those actually mapped with the Watson data, (iii) a BLAST search of unmapped ref-TRs from the Watson analysis against the Watson reads to determine how many mappings were missed, (iv) an analysis of indels deposited in dbSNP (63) by the authors of the Watson sequencing paper in order to determine which are coincident with the VNTRs we report, and (v) analysis to determine the degree of observed Mendelian inheritance in VNTRs in two family trios from the 1000 Genomes Project (64,65).

#### Simulation

Three simulated 454 read sets were generated, equivalent in size and read length characteristics to the Watson data set. An additional three sets were produced from these which contained simulated sequencing errors. Similarly, six simulated Illumina 100 nt reads sets were generated, three exact and three with errors, and all with coverage equivalent to the Watson data set. All 12 sets were mapped, using a minimum flank length of 20 nt, to a modified reference set which contained 1118 artificial VNTR loci (approximately 0.5% of the total reference TRs). Mapped locations were then compared to the origins of the reads and called VNTRs were compared to the artificial VNTRs. Table 1 gives average accuracy measures for all 12 sets and Supplementary Tables S2-S7 show results for one representative run each from the unmodified and modified read sets.

**Table 1.** VNTRseek accuracy, minimum flank length 20

					Genotype calling							
Read	Read mapping		Reference TR mapping		Unmodified TR		Homozygous VNTR			Heterozygous VNTR†		
Set	Sen	Spec	Sen	Spec	Sen	Spec	Sens	Spec	PPV	Sens	Spec	PPV
454 Exact (avg. 261 nt)	97.5%	99.6%	96.9%	99.2%	97.7%	100%*	95.8%	100%*	96.3%	84.2%	100%*	91.6%
454 Errors (avg. 261 nt)	90.1%	99.5%	94.7%	99%	93.7%	99.9%	91.9%	100%*	91.8%	76.6%	100%*	85.8%
Illumina Exact (100 nt)	94.5%	99.5%	96.8%	99.6%	95.0%	100%*	93.6%	100%*	98.1%	83.1%	100%*	86.5%
Illumina Errors (100 nt)	70.4%	97.7%	78.1%	97.4%	67.6%	100%*	64.8%	100%*	94.2%	47.3%	100%*	74.5%

Average accuracy measures for 12 simulated read sets, three each for two technologies (454 and Illumina) generated from the reference genome (Exact) and three each obtained by introducing errors into exact reads (Errors). Read Mapping is the accuracy of assigning reads to the correct reference TRs. Reference TR Mapping is the accuracy with which reference TRs were assigned reads. Genotype Calling is the accuracy of calling unmodified reference TRs and homozygous VNTRs in a modified reference set where 1118 randomly selected reference TRs (approximately 0.5% of the total) were modified by adding or subtracting one or two pattern copies, and the accuracy of calling heterozygous VNTRs where the unmodified reference set was used and reads were selected equally from two chromosome sets, one exact and one modified to match the modified references. PPV is positive predictive value, the fraction of called VNTRs that were correct. Typical data are shown in Supplementary Tables S2-S6. *Specificity for unmodified TR calling and VNTR calling is slightly less than 100%. †Heterozygous VNTR values for Illumina reads obtained by combining three data sets into one in order to obtain enough ref-TR loci spanned by at least two reads in both the modified and unmodified chromosome sets.

An additional 12 simulated read sets were produced and mapped to the modified reference set using a minimum flank length of 10 nt. Average and representative results are shown in Supplementary Tables S9-S13.

For reads mapped with a minimum flank length of 20 nt, accuracy was high for all measures, except sensitivities for the simulated Illumina 100 nt reads with errors. This was primarily due to the inability of TRF to detect relatively short TR arrays with errors in many of the reads (see Supplementary Table S3). Because the negative set for VNTR calling was much larger than the positive set, the positive predictive value (PPV) or fraction of VNTR calls that are correct, is an important measure. From Table 1, for the read sets with errors and testing with homozygous VNTRs, the PPVs were 91.8% (454) and 94.2% (Illumina) which means approximately 1 in 12 (454) and 1 in 17 (Illumina) called VNTRs were wrong. Examining a typical case for homozygous testing (Table S5), and subdividing the PPV into values for singletons and indistinguishables, for 454 reads with errors, approximately 1 in 21 calls were wrong for singletons, whereas approximately 1 in 2 calls were wrong for indistinguishables. Other cases were similar. These results give further evidence for highlighting singleton and indistinguishable classes in the output. Sensitivity for VNTR detection under heterozygous conditions was lower compared to homozygous conditions, however PPVs were still moderately high, 85.8% (454) and 74.5% (Illumina).

For the reads mapped with a minimum flank length of 10 nt, sensitivity and specificity were similar to those obtained with a minimum flank length of 20 nt, however, the PPV values were slightly lower. For the shorter Illumina reads, the number of ref-TRs with mapped reads increased significantly, as expected, because more of the read length was available to span a TR. On balance, the results suggest that the shorter minimum flank length should be used with shorter reads.

#### Expected mappings for different read lengths

VNTRseek requires reads that span a TR array with sufficient non-repetitive flanking sequence to establish correct mappings and copy numbers. Supplementary Table S1 shows the expected proportion of human reference TRs that can be mapped using different read lengths and coverages, as calculated by formulas that determine the probability that a TR of a given array length will be spanned by at least one or two randomly placed reads, assuming all reads have a fixed length (see Supplementary Material). Supplementary Table S1 can be used as a guide when considering potential data sets. 100 nt reads are expected to map reads to 45% to 73% of the references when coverage is 5 or above and 20 nt is the minimum required flank length. 250 nt reads are expected to map reads to 88% to 94% of the references under similar conditions.

#### Expected mappings in the Watson data

We calculated the expected number of ref-TRs that would have at least one mapped read for a data set comparable in size to the Watson data and compared that to the results from the Watson mapping. Two methods were used, the probability formula and a simulation. The formula applies to *fixed length* reads. For the simulation, we used the actual locations and array lengths of the ref-TRs and determined how many were covered, on average, by randomly placed reads with *lengths drawn from a normal distribution* similar to that of the Watson data. The two methods gave very similar results and are compared with the performance of VNTRseek on the Watson data in Figure 2. Notice that the expected frequencies are significantly higher than the observed frequencies for the real data. We attribute this primarily to three factors, failure to map indistinguishable ref-TRs (approximately 13% of the unmapped references) perhaps because reads that map with equal scores to more than one reference are discarded, failure of TRF to find read-TRs with too many errors, as observed in the simulation results (Supplementary Tables S3 and S9), and underrepresentation of repeat regions in whole genome sequencing data as observed by others using *microsatellite* VNTR discovery programs (36,38,40).

#### BLAST search

60,843 ref-TRs were unmapped in the Watson analysis. Of those 11,261 had reads assigned, but lost those reads to other references due to better profile or flank alignments, or because reads were discarded due to ties. The remaining 49,582 ref-TRs were used as queries against two BLAST databases built from the Watson reads. Queries included 20 nt of flanking sequence on each side of the tandem array (the minimum required for VNTRseek to map a read). The first database contained all the Watson reads. 2302 ref-TRs had hits to the database for which an HSP contained at least 19 nt of flanking sequence on each side of the query sequence. These ref-TRs constitute 1% of the total ref-TRs and 5% of the ref-TRs never assigned reads. The second database contained only Watson reads for which TRF reported a TR. In this smaller set, which consisted of the reads actually processed by VNTRseek, 968 ref-TRs had hits as above (0.4% of the total ref-TRs and 2% of the ref-TRs never assigned reads). These results suggest that in the vast majority of cases where VNTRseek failed to assign reads to a reference, spanning reads were not present in the data.

#### Watson indels in dbSNP

A comparison was made between the indels deposited in dbSNP by the authors of the Watson sequencing paper (52) and the VNTRs reported by VNTRseek in the Watson genome (Figure 3). Indel loci overlapped with 1167 ref-TRs and had a reported gain or loss equivalent to one or more repeat units, suggesting that they are VNTRs. However, only 260 coincided with the 752 VNTRs detected by VNTRseek (with 99.2% concurrence on indel size between the two analyses). Further examination of the 907 putative dbSNP indels not detected by VNTRseek revealed that 698 (77%) were deletions that resulted in less than 1.9 copies of the TR pattern remaining in the array, at which point TRF is unable to detect a repeat. An additional four were deletions that extended too far into the flanking sequence to allow detection. A BLAST search was conducted with the remaining 205 indels against sequence databases containing all the reads or only those for which TRF reported a TR, as described above. 68 (7%) were found to have at least two hits in all the reads and 19 (2%) were found to have at least two hits in the reads passing the TRF filter and could have been detected by VNTRseek. Of the 19, the majority of those were not detected because they contained too many errors in the flanks. Thus, the primary reasons that the dbSNP entrees were not detected by VNTRseek were (i) the loci no longer look like TRs after a deletion, and (ii) two spanning reads containing a TR detectable by TRF were not available in the data.

#### 1000 Genomes trios

A comparison of genotype consistency with regard to Mendelian inheritance was made in two family trios with high coverage (68x-81x) from the 1000 Genomes project. A VNTR locus was considered if the data for an individual provided support for two alleles. From 46% to 52% of the total VNTR loci in each individual met this criterion (contrasting with the lower coverage Watson genome for which only 17% met the criterion). Results are shown in Table 2. Several hundred bi-allelic loci were shared among each trio's members and a much smaller subset exhibited different genotypes in each individual, required so that Mendelian inheritance could be unambiguously tested. Nonetheless, among all the bi-allelic loci in common, only one locus in the Utah family was inconsistent with Mendelian inheritance. (Individual vcf files for trio members and files with bi-allelic alleles in common are available in Supplementary Material.)

**Table 2.** Mendelian inheritance of VNTRs in 1000 Genomes trios

Utah family						Nigerian family					
Daughter	Mother	Father	Loci		Incon-	Daughter	Mother	Father	Loci		Inconsistent
NA12878	NA12892	NA12891	All	Diff	sistent	NA19240	NA19238	NA19239	All	Diff	
1241	1327	1402	274	20	1	1963	1979	1956	437	55	0

Shown are the number of VNTR loci for which two alleles were supported in each individual (sum of Same/Diff and Diff/Diff as in Table 3 C), number of loci in common for the trio (All), the subset of loci in common for which all three have different genotypes (Diff), and the number of loci inconsistent with Mendelian inheritance. The inconsistency count applies to all the loci in common, although the subset of loci for which all the family members are heterozygous AND have different genotypes provides the strongest test that VNTRseek is not systematically mis-assigning alleles from different loci to the same locus. Note that the VNTR loci with two alleles supported ranged from 46% to 52% of the total VNTRs in these individuals (data not shown). This contrasts with the lower coverage Watson genome in which 17% of loci exhibited two alleles.

### Indistinguishable ref-TRs

Profile and flank alignments used to compare the ref-TRs to themselves identified 13,941 (6%) that are indistinguishable. Of these, 57% occur in clusters of four or fewer references. Supplementary Figure S8 gives the distribution of indistinguishable cluster sizes.

Following initial testing with simulated data, it was determined that some TRs filtered from the reference set were causing artifactual detection of VNTRs because reads spanning those TRs were mapping to remaining ref-TRs that belonged to the same indistinguishable family. A series of six simulated data mappings were used to detect unfiltered ref-TRs which produced artifacts and 365 TRs (0.16%) were subsequently removed from the reference set. This reduced the number of false positive VNTRs from 108 (with 40 singletons) to an average of 34 (8 singletons) when tested on an additional three simulated data sets. Perfomance results reported above are for mappings after the removal of the 365 ref-TRs.

### Individual genome results

#### Mapping and VNTR calls

Table 3 sumarizes the VNTRseek results for the Watson genome. Among the references, 74% were assigned at least one read. (Note that nearly 6% of the references have array lengths plus flanks over the average Watson read length and so were unlikely to be assigned reads.) Of the mapped references, 78% had at least one allele supported. 752 VNTR loci were detected, yielding a total of 759 variant alleles. For the VNTR loci, only 17% had two alleles supported. Of the Watson VNTRs, 4% were indistinguishable and we cannot be confident about the VNTR calls for those references.

**Table 3.** Watson VNTRseek results

A. Mapping					B. Mapped reference results				
	Total	After filters	Mapped	%	Number of reads mapped		At least one allele supported	By reference category	
Ref-TRs	1,188,939	230,306	169,463	74	≥ One	≥ Two		Singleton	Indist.
Read-TRs	13,080,867	4,826,849	532,960	11	169,463	131,855	131,707	164,080	5,383
Reads	74,198,831	2,925,732	525,748	18	100%	78%	78%	97%	3%

C. VNTR results									
			Alleles supported						
			One	Two					
			⋆	•	•	By reference category			
		Total	Diff	Same/Diff	Diff/Diff	Singleton	Indist.		
		752	627	118	7	720	32		
		100%	83%	16%	1%	96%	4%		

A. Input data and data after filtering the reference set (for quality, common interspersed repeats, redundancy and pattern size) and the read set (for pattern size and sufficient flanking sequence); B. Counts and percentages of mapped references that were assigned at least one read, at least two reads, had at least one allele supported, and were either singleton or indistinguishable. An allele was *supported* if at least two reads were assigned to the ref-TR and they agreed on the pattern copy number. A ref-TR is indistinguishable if it is highly similar in both profile and flank alignments with another reference. All others are singletons. C. Counts and percentages of total VNTRS, number of alleles supported and reference category. For one allele supported, ‘Diff’ means the number of copies is different from the reference. These are inferred VNTRs because the reference is assumed to be correct, i.e. not an artifact. For two alleles supported, ‘Same/Diff’ means one allele has the same number of copies as the reference; ‘Diff/Diff’ means neither does; these are observed VNTRs because two alleles are observed. ⋆ Inferred VNTR • Observed VNTR

Supplementary Table S14 gives results for the Khoisan genome. Among the references, 82% were assigned at least one read and of those, 92% had at least one allele supported. 2572 VNTR loci were detected yielding a total of 2693 variant alleles. Among the VNTR loci, six had more than two alleles reported, which is unexpected. Only two of these loci were indistinguishable, suggesting that the reference set contains additional indistinguishable references that are miscategorized. 36% of the VNTR loci had two alleles supported. This is intermediate between the rates for the Watson data set (17%) which has approximately half the coverage and the 1000 Genomes trios data sets (nearly 50%) which have four to five times the coverage. The distribution between singleton and indistinguishable VNTRs was similar to the Watson results.

Supplementary Table S15 gives the results for the NA12878 genome. Among the references, 87% were assigned at least one read and of those, 98% had at least one allele supported. 2659 VNTR loci were detected yielding a total of 2812 variant alleles. 15 of the VNTR loci had more than 2 alleles reported and 6 of those were indistinguishable. 48% of the VNTR loci had two alleles supported.

(Watson, Khoisan and NA12878 vcf files are available in Supplementary Data.)

##### VNTR characteristics.

Figures 4 and 5 present characteristics of the Watson VNTRs, including distributions across pattern sizes and array lengths, and the frequency of loss or gain of copies *relative to the reference*. Supplementary Figures S2 and S3 show VNTR characteristics for the Khoisan data and Supplementary Figures S4 and S5 show VNTR characteristics for the NA12878 data. VNTRs were detected with pattern size ranging from 7 nt (the lower limit in this study) to 84 nt (Watson) and 105 nt (Khoisan). The majority of the variants had pattern sizes of 30 nt or smaller (Watson 81%, Khoisan 74%, NA12878 76%).

Loss or gain of a single copy relative to the reference was the most commonly detected variant and loss of copies was more frequent overall. Loss can be detected more frequently than gain by a fixed read size for any TR, and a bias toward detection of loss is introduced by the limited read size (e.g. gain of a 100 nt pattern is not possible in a read of length 250 nt when there are already two copies present). However, all three individual genomes examined show an excess of copy gain up to array lengths of about 50 nt where the difference in detection rates should be negligible (Figure 4 and Supplementary Figures S6 and S7). The Watson data show an abrupt shift to copy loss at 50 nt (Figure 4), whereas the Khoisan and NA12878 data show a gradual shift from neutral between 50 and 60 nt to loss at higher lengths, as expected due to an inability to detect copy gain at larger array and pattern sizes with reads of limited length.

The excess of copy gain observed up to 50 nt could have several explanations: the reference may contain artifactual errors which reduce the number of copies at shorter array lengths; loss in shorter arrays may cause the copy number to fall below two so that the locus no longer looks like a TR; and sequencing errors in shorter arrays may cause TRF to fail to detect the repeat. Another possibility is that there are different processes controlling gain and loss at different array lengths. Analysis of microsatellites mutations in a large human population has shown that shorter alleles tend to increase in length and longer alleles tend to decrease in length (66). The reason for the abrupt shift in the Watson data is not known and is inconsistent with the Khoisan and NA12878 data. Note that loss or gain, as used here, does not indicate direction of mutation, if any, in these individuals because that cannot be established without knowledge of the alleles in the parents.

### Program usage

**Figure 1.**
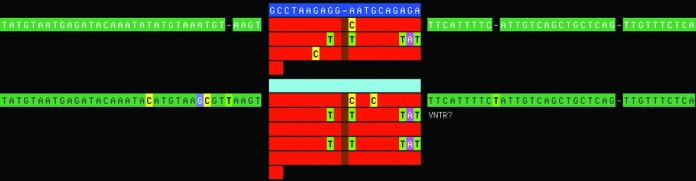
VNTR. Watson read-TR (bottom) mapped to a ref-TR (top) from Chr 15:23,215,373. The number of copies, pattern motifs and motif order differ between the two, conditions which favor profile-based alignment. Blue—consensus pattern of ref-TR; red—multiple alignment of individual copies within a repeat, red matches the consensus, differences are shown explicitly; order of copies vertically matches order in the tandem array; light blue—consensus pattern of read-TR, here with no differences from the ref-TR consensus; green—flanking sequence with differences; not all available flanking sequence is shown due to page width limitation. Vertical gap due to insertion in another repeat (not shown).

VNTRseek is a combination of C source code and Perl scripts which interact with a MySQL database created as part of the processing. Input is a set of FASTA or FASTQ files holding the subject reads and TR reference set data. Output consists of web pages which summarize the results of each program step; sortable tables which list the characteristics of each mapped ref-TR, and each called VNTR; visualizations of read-TR to ref-TR alignments (e.g. Figure 1 ); Latex output of mapping statistics (Table 3 and Supplementary Tables 14 and 15; and two VCF format files, one for VNTRs detected and the other for all genotyped ref-TRs whether they are found to be variable or not. VCF files contain URL links to the alignment visualizations. All source code, instructions and the human TR reference set data are available at http://orca.bu.edu/vntrseek/.

**Figure 2.**
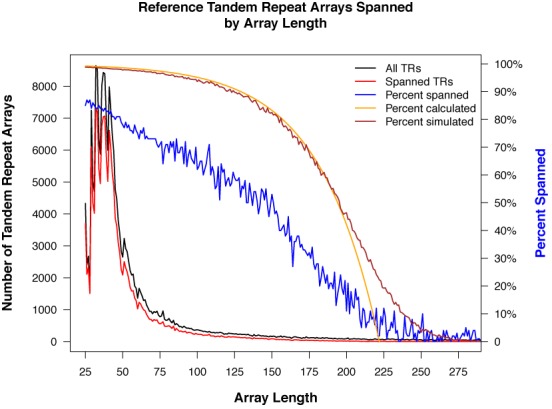
Reference TRs spanned by at least one read, expected versus Watson observed. ‘All TRs’ is the number of reference TR loci at each array length (combined length of all copies in the TR). ‘Spanned TRs’ have at least one read mapped by VNTRseek analysis. ‘Percent spanned’ is the ratio of spanned loci to all loci. ‘Percent calculated’ is an expected value for percent spanned derived using a probability formula for autosomal TR arrays and a fixed read length equal to the average Watson read length. ‘Percent simulated’ is an expected value for percent spanned obtained as the average number of reference TRs covered by randomly placed reads, in 50 simulations, with read lengths drawn from a normal distribution similar to that of the Watson reads. Differences between simulated and calculated percentages are due to occurrence in the simulations of longer read lengths and reference TRs on the X and Y chromosomes (not the autosomes). Observed results suggest a significant underreporting of spanned loci. This is attributed primarily to failure to map indistinguishable TRs, failure of TRF to detect TRs in reads with too many mutations or errors, and undersampling of the genome in repeat rich regions.

**Figure 3.**
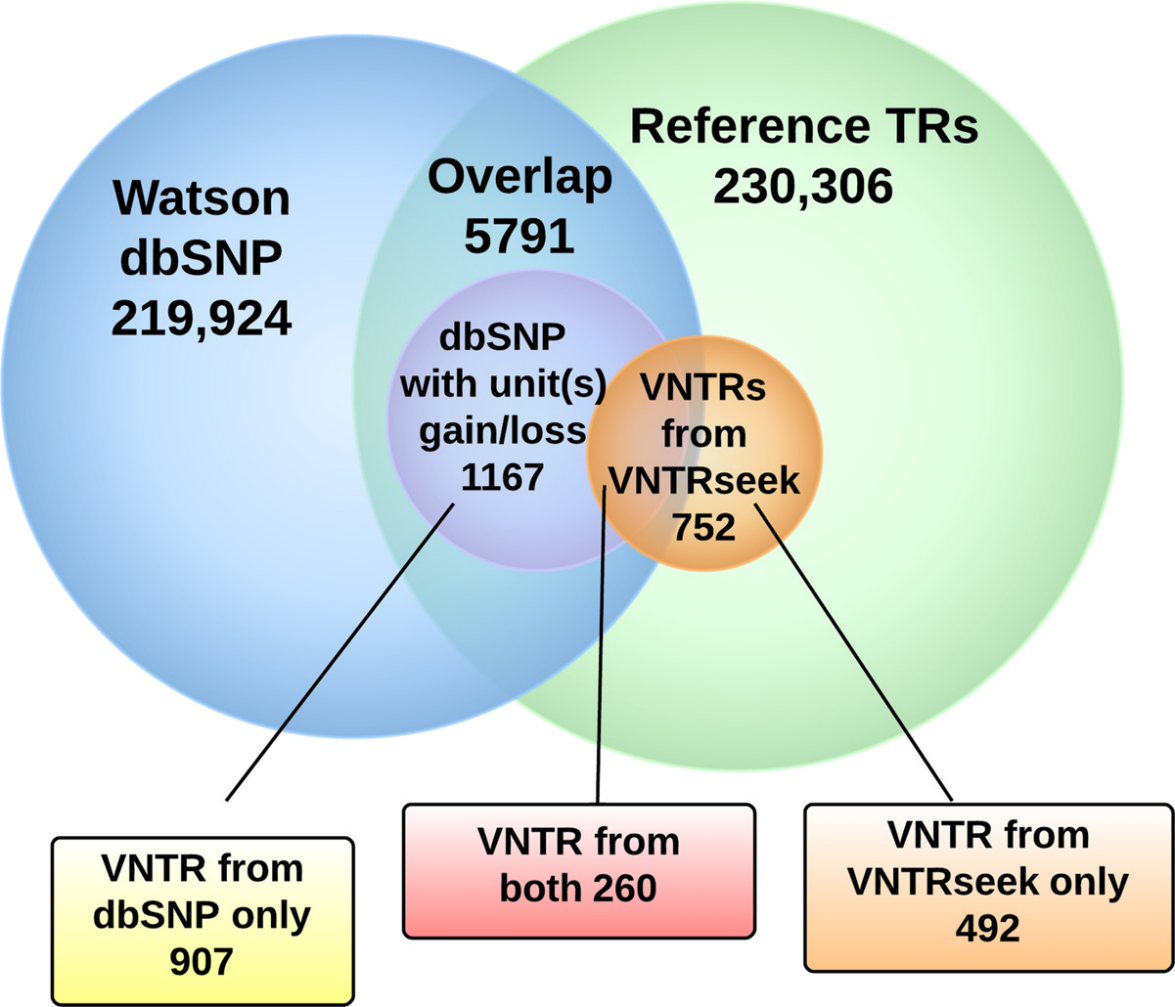
Comparison of VNTRseek detected VNTRs to Watson indels in dbSNP.

**Figure 4.**
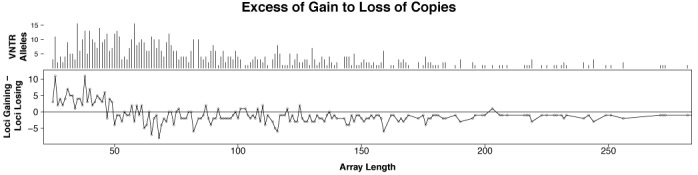
Watson VNTRs. Top: distribution of VNTRs by array length in the reference. Bottom: difference between number of loci that show copy gain and number that show copy loss, relative to the reference. More VNTRs show gain than loss at array lengths under 50 nt (trace above line). Abruptly, loss of copies becomes more common at longer array lengths. (Omitted from the graph are 15 VNTRs with reference array length longer than 282 nt.)

**Figure 5.**
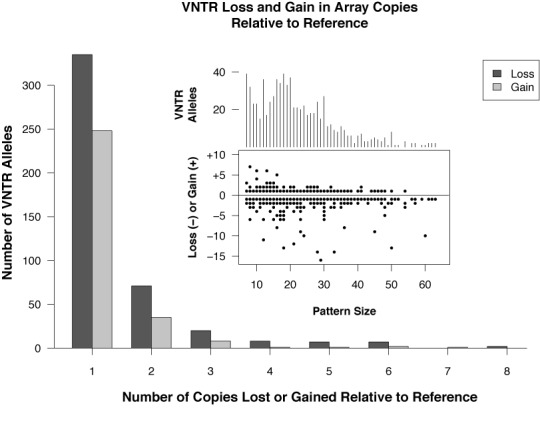
Watson VNTRs. Large graph: distribution of copy loss or gain relative to the reference. Singe copy change is by far the most commonly detected VNTR allele. Loss of copies overall is more frequent than gain. *Inset top:* distribution of VNTRs by pattern size. *Inset bottom:* number of copies gained or lost, by pattern size. Note that frequency for each gain or loss is not shown, only occurrence. Data are for 759 variant alleles from 752 reference TRs called as VNTRs. (Omitted from large graph are 13 VNTRs with loss/gain greater than 8. Omitted from offset bottom are two VNTRs with loss greater than 16 copies. Omitted from both insets is one VNTR with pattern size = 84 nt.)

## DISCUSSION

In order to facilitate discovery of VNTRs, we have developed the VNTRseek software which identifies internal copy number variation at minisatellite TR loci. The repetitive nature of TRs makes them difficult genomic features to map accurately. VNTRs are indels with respect to the reference genome, but in the absence of spanning reads, may not yield the split-read signature common to indels in non-repetitive sequence. Inexact pattern copies may also be rearranged in a VNTR and could cause mappings that look like indels even when no copy number difference exists.

Matching of consensus patterns for minisatellites is more complicated than for microsatellites. In the latter case, the small patterns can be enumerated into a limited number of classes for both the references and the reads. For minisatellites, with larger patterns, there are too many patterns to enumerate and small mutational or sequencing error differences would cause mismatch between read and reference patterns. We have chosen to detect matches with a combination of consensus sequence and profile alignments.

Mappings are confirmed with flanking sequence alignment, a technique also used in microsatellite VNTR detection programs. In our analyses, mapping results were sensitive to the length of read-TR flanking sequences used for alignment. All flanking sequences were at least 20 nt long and in the Watson and Trios data, lengths were unrestricted and flanks extended to the ends of the reads. For the much longer Khoisan reads, flanks were limited to 50 nt. Without this limit, the number of Khoisan VNTRs reported was reduced by two-thirds. This was most likely due to our stringent flank score threshold and the likelihood of sequencing errors in the lower quality ends of the Khoisan reads. In the future, we will likely choose a 50 nt upper bound on flank lengths.

Indistinguishable references, which comprise 6% of our reference set, are likely to cause incorrect VNTR calls and are flagged in VNTRseek output. Microsatellite VNTR detection programs do not explicitly identify such references, but some do exclude references that are mapped by an unusually high number of reads. However, a majority of minisatellite indistinguishable families contain from two to five members and this low number suggests that variation from average spanning read coverage would be an insufficient filter for minisatellites and may be also for microsatellites.

Our reference filtering sometimes removed all but one member of an indistinguishable family and that member, miscategorized as a singleton, can cause incorrect and unflagged VNTR calls. An *ad-hoc* method for identifying these references by mapping simulated reads removed several hundred problematic references and significantly reduced false positive singleton VNTR calls. But, a more robust method is required and we are now exploring mapping the unfiltered references to the filtered references to identify indistinguishables.

Validation testing of the software yielded high nominal measures of accuracy on simulated data. Actual mapping on the Watson test data was significantly lower than expected, a difference that may be due to the presence of indistinguishables, loss of TRF detection of TRs in reads with a high number of mutations or sequencing errors and reads from loci where deletion has left less than two pattern copies, and underrepresentation of reads from repetitive genomic regions. In support of this last possibility, use of BLAST to search for reference matches in the Watson reads found matches to only 5% of the unmapped references, suggesting that there were no spanning reads for the other 95%. The significant number of reference TR loci represented in dbSNP that have undergone deletion to fewer than two pattern copies in the Watson genome suggests that software specifically tailored to find such ‘truncated’ TRs would be useful. VNTRseek cannot currently detect them.

Analysis of two 1000 Genomes family trios found 20 and 55 heterozygous loci in which inheritance could be unambiguously tested. In all cases but one, alleles were consistent with Mendelian inheritance.

Analysis of the Watson and Khoisan genomes yielded 3324 VNTRs. Analysis of the family trios yielded nearly 20,000 VNTRs. Excluding overlaps, we have identified 7378 putative VNTRs.

We observed an anomalous tendency for copy number increase over decrease in shorter TR arrays in the Watson, Khoisan and NA12878 genomes where the difference should be negligible. This is not yet explained, but may be due to sequence loss of shorter TRs, a tendency for short alleles to gain length, program bias, or artifactual errors in the reference at TR loci, which, like many classes of repeats, present difficulties in assembly. Over time, as more VNTRs are detected and confirmed, we expect that reference errors will become apparent and addition of variants to the reference set will improve the mapping.

VNTRseek run time was 6.6 h for the Watson genome, 19.6 h for the Khoisan genome and 48.2 h for the NA12878 genome on a 16 processor workstation with 128 GB of RAM memory. We are in the process of streamlining the program. In particular, TRF analysis takes up from one-third to one-half of the run time and we have plans to accelerate TRF with new bit-parallel alignment algorithms (67).

We expect VNTRseek will be useful for identifying common and rare minisatellite VNTR loci in the human and other genomes. The resulting data set of genome-wide VNTR occurrence will be applicable to genotype/phenotype association studies and can be expected to lead to a more complete understanding of VNTR dynamics.

## SUPPLEMENTARY DATA

Supplementary Data are available at NAR Online.

SUPPLEMENTARY DATA
