# Supplementary material for: VNTRseek—a computational tool to detect tandem repeat variants in high-throughput sequencing data
Source: Nucleic Acids Res. 2014 Jul 23;42(14):8884–94. doi: 10.1093/nar/gku642 (PMC4132751; doi:10.1093/nar/gku642)
Supplement: SUPPLEMENTARY DATA [file supp_42_14_8884__index.html]

VNTRseek—a computational tool to detect tandem repeat variants in high-throughput sequencing data — VNTRseek—a computational tool to detect tandem repeat variants in high-throughput sequencing data — SUPPLEMENTARY DATA 

# VNTRseek—a computational tool to detect tandem repeat variants in high-throughput sequencing data

## SUPPLEMENTARY DATA

**Files in this Data Supplement:**

- SUPPLEMENTARY DATA
