# Supplementary material for: VNTRseek—a computational tool to detect tandem repeat variants in high-throughput sequencing data
Source: Nucleic Acids Res. 2014 Jul 23;42(14):8884–94. doi: 10.1093/nar/gku642 (PMC4132751; doi:10.1093/nar/gku642)
Supplement: SUPPLEMENTARY DATA [file supp_gku642_nar-00437-n-2014-File011.zip › Supplementary Material.pdf]

Published online 00 XXXXX 2014 *Nucleic Acids Research, 2014, Vol. XX, No. XX* **1–9**
doi:10.1093/nar/gkn000

Supplementary Material
VNTRseek – A Computational Tool to Detect Tandem Repeat Variants in High-Throughput Sequencing Data

Yevgeniy Gelfand, Yozen Hernandez, Joshua Loving, Gary Benson

Calculation of expected number of spanned TR loci. (See Figure S1.) We assume that TR loci are far enough apart so that the probability of a read spanning more than one locus is negligible. Let:

- r be the fixed read length (here equal to 261).
- a be a given TR array length.
- f be the required flanking sequence length.
- $s = r - (a + 2f) + 1$ be the size of the interval $5'$ to TR-ref array in which a read can start and be said to span the array. See Figure S1.
- n_r be the number of reads
- $g_d = 2g_A + g_{XY}$ be the *male* diploid genome length where g_A and g_{XY} are genome lengths for, respectively, the autosomal chromosomes (two copies) and the X and Y chromosomes (single copies).
- $p = n_r/g_d$ be the probability that a read starts at any given diploid genome location. Without loss of generality, we say that a read starts at the leftmost ($5'$) genomic location to which it maps.
- $1-p$ be the probability that no read starts at the given location.

In a diploid genome, for each TR locus on an autosome, there are two intervals of size s that can be hit in order for the locus to be spanned, one on each copy of the chromosome. We can think of those intervals as a single interval of size $2s$. The probability that a locus is *not* spanned in either copy of the genome is the probability that no read starts at any of the 2s positions:

$$P_0 = (1-p)^{2s}$$

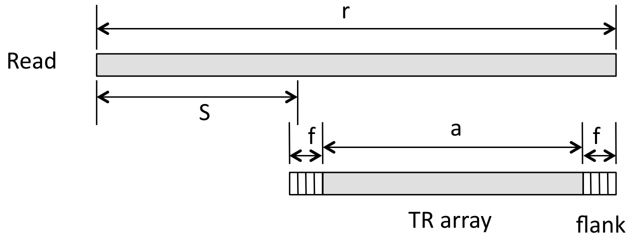

Figure S1. S is the length of the genomic region where a read of fixed length can start and span the TR array.

and the probability that a locus is spanned *exactly once* across both copies is

$$P_1 = 2sp(1-p)^{2s-1}$$

where the $2s$ term is the possible starts for the spanning read, p is its probability, and $(1-p)^{2s-1}$ is the probability that the remaining $2s-1$ positions are not read starts. The corresponding equations for the X and Y chromosomes, which occur only once in a male genome, are

$$P_0^{X,Y} = (1-p)^s \text{ and } P_1^{X,Y} = sp(1-p)^{s-1}.$$

The probability that a TR locus is spanned by *at least one or two* reads is:

$$P(\text{Spanned at least once}) = 1 - P_0$$
$$P(\text{Spanned at least twice}) = 1 - P_0 - P_1.$$

Read Length (nt)	Minimum Flank Length					
	20			10		
	Coverage			Coverage		
	T	30	5	T	30	5
50	0	0	0	10	8 / 5	3 / 0
75	25	20 / 14	7 / 1	69	67 / 64	45 / 22
100	73	71 / 67	45 / 21	82	81 / 80	67 / 45
150	87	87 / 86	75 / 54	89	89 / 88	82 / 66
250	94	94 / 93	88 / 75	95	94/ 94	90 / 80

Table S1. Expected fraction, in percent, of human reference TRs that will be spanned by at least one read and at least two reads for various read lengths in terms of genome coverage and minimum flanking sequence length. ”T” is the theoretical fraction spanned based only on reference array length. With Illumina 100 nt reads, a coverage of 30, and a minimum flank length of 20 nt, 71% of the references are expected to be scanned by at least one read and 67% are expected to be spanned by at least two reads. With 250 nt reads, under the same conditions, 94% of the references are expected to be spanned by at least one read and 93% by at least two reads.

© 2014 The Author(s)
This is an Open Access article distributed under the terms of the Creative Commons Attribution Non-Commercial License (http://creativecommons.org/licenses/by-nc/2.0/uk/) which permits unrestricted non-commercial use, distribution, and reproduction in any medium, provided the original work is properly cited.

Randomly Generated Reads				
Read Set	Reference TR Spanning		Other TR Spanning	
	Generated	Correctly Mapped	Generated	Incorrectly Mapped
454 Exact (avg. 261 nt)	855,782	834,651	1,607,836	7,050
	100%	97.5%	100%	0.4%
454 Errors (avg. 261 nt)	855,782	771,335	1,654,924	7,575
	100%	90.1%	100%	0.5%
Illumina Exact (100 nt)	224,533	212,197	439,832	2,025
	100%	94.5%	100%	0.5%
Illumina Errors (100 nt)	223,290	157,452	439,447	10,232
	100%	70.5%	100%	2.3%

Table S2. Read Mapping Accuracy, Minimum Flank Length 20. Results for four typical simulated read sets generated from the reference genome and mapped back to the reference TRs. Two sets are 454 type reads, two are Illumina type reads. Reads in the Exact sets exactly match the reference while reads in the Errors sets contain simulated sequencing errors. Reference TR Spanning reads are those that spanned the locus of a reference TR including at least twenty nucleotides of flanking sequence on each side. Correctly mapped means the read was mapped to the correct reference TR. Other TR Spanning reads are those that contained a spanned TR, but not a reference TR. Incorrectly mapped in this group means the read was mapped to a reference TR when it should not have been mapped to anything. Sensitivity is the percentage in column 3. Specificity is 1−the percentage in column 5. The 454 reads in all the simulations had an average length of 261 nt. Illumina reads in all the simulations had a length of 100 nt.

Reference TR Spanning Reads Not Correctly Mapped				
Read Set	All	Mapped Incorrectly	Failed TRF	Other
454 Exact	21,131	462	545	20,124
454 Errors	84,447	783	33,281	50,383
Illumina Exact	12,336	66	379	11,891
Illumina Errors	65,838	86	52,609	13,143

Table S3. Fate of Reference TR Spanning Reads Not Mapped Correctly, Minimum Flank Length 20. Results for four typical simulated read sets generated from the reference genome and mapped back to the reference TRs. A very small number of reads mapped to the wrong reference. In the reads with errors, reads were commonly discarded because the errors affected detection of the repeat by TRF. This was especially true in the shorter Illumina type reads, because the spanned TRs contained fewer copies and were more easily disrupted. Other reasons for discarding a read were a) ties – the read-TR mapped to more than one reference with equal score, b) multiple loci – different read-TRs in the same read mapped to two references that were not close enough together to be spanned by the read or mapped to three or more references no matter their spacing, c) the read was classified as a PCR duplicate, and d) TR profile or flank scores failed to meet the thresholds.

Reference TRs							
Read Set	With Spanning Reads	With Mapped Reads			Without Spanning Reads	With Mapped Reads	
		Only Correct	Other	None		None	Any
454 Exact	209,519	203,074	1,923	4,522	20,787	20,639	148
	100%	96.9%	0.9%	2.2%	100%	99.3%	0.7%
454 Errors	209,519	198,365	2,783	8,371	20,787	20,597	190
	100%	94.7%	1.3%	4.0%	100%	99.1%	0.9%
Illumina Exact	115,821	112,086	812	2,923	114,485	113,981	504
	100%	96.8%	0.7%	2.5%	100%	99.6%	0.4%
Illumina Errors	115,693	90,300	6,698	18,695	114,613	111,647	2,966
	100%	78.1%	5.8%	16.2%	100%	97.4%	2.6%

Table S4. Reference Mapping Accuracy, Minimum Flank Length 20. Results for four typical simulated read sets generated from the reference genome and mapped back to the reference TRs. Only those references with at least one spanning read were considered (88% of the references in the 454 reads and 50% of the references in the Illumina reads, both approximately as expected from Table S1.). Sensitivity is the percentage shown in column 3. Specificity is the percentage shown in column 7. Lower sensitivity in the Illumina reads with errors was primarily due to discarding reads in which TRF did not detect the repeat.

Generated Homozygous VNTRs					Other Reference TRs						
Read Set	2-span	Called		Not Detected	2-span	Called		Other	Called		VNTR PPV
		Correct	Incorrect			Correct	VNTR		No	VNTR	
454 Exact	913	874	1	38	189,592	185,210	19	39,801	39,785	16	–
	100%	95.7%	0.1%	4.2%	100%	97.7%	0.0%	100%	100.0%	0.0%	96.0%
454 Errors	913	829	1	83	189,592	177,700	54	39,801	39,779	22	–
	100%	90.8%	0.1%	9.1%	100%	93.7%	0.0%	100%	99.9%	0.1%	91.5%
Illumina Exact	315	302	0	13	63,620	60,421	3	166,371	166,368	3	–
	100%	95.9%	0.0%	4.1%	100%	95.0%	0.0%	100%	100.0%	0.0%	98.1%
Illumina Errors	282	177	0	105	63,200	42,730	2	166,824	166,815	9	–
	100%	62.8%	0.0%	37.2%	100%	67.6%	0.0%	100%	100.0%	0.0%	94.1%

Table S5. Genotyping Accuracy for Homozygous Data, Minimum Flank Length 20. Results for four typical simulated read sets generated from the reference genome and mapped back to the reference TRs. 1118 randomly selected reference TRs (approximately 0.5% of the total) were modified by adding or subtracting one or two pattern copies. Only those references with at least two spanning reads were considered (the minimum required to call an allele) when determining genotype calling sensitivity. Sensitivity of VNTR calling is the percentage in column 3. Specificity of VNTR calling is the ratio of all other reference TRs not called as VNTRs (230306 - column 2 - column 8 - column 11) to all other reference TRs (230306 - column 2) and is essentially 100% in all cases. Given the large negative set size, an important measure is positive predictive value (PPV), the ratio of true VNTR calls to all VNTR calls (column 12). In the 454 read set with errors, 8.5% of the VNTR calls were incorrect (approximately 1 out of 12). When subdivided, this corresponds to approximately 1 in 21 (40/840) incorrect calls for singletons and 1 in 2 (36/65) incorrect calls for indistinguishables. In the Illumina read set with errors, 5.9% of the VNTR calls were incorrect (approximately 1 out of 17). When subdivided, this corresponds to approximately 1 in 17 (11/183) incorrect calls for singletons and 0 incorrect calls for indistinguishables (0/5). Sensitivity of unmodified TR calling is the percentage in column 7. Specificity of unmodified TR calling is the percentage in column 10.

Generated Heterozygous TRs					Other Reference TRs			
Read Set	2-span both alleles	Called Heterozygous				Called Heterozygous		PPV
		Correct	Incorrect	No	All	No	Yes	
454 Exact	323	274	2	47	229,983	229,966	17	-
	100%	84.8%	0.6%	14.5%	100%	100%	0%	94.2%
454 Errors	323	250	2	71	229,983	229,940	43	-
	100%	77.3%	0.6%	21.9%	100%	100%	0%	85.3%
†Illumina Exact	77	64	0	13	230,229	230,219	10	-
	100%	83.1%	0%	16.9%	100%	100%	0%	86.5%
†Illumina Errors	74	35	0	39	230,232	230,220	12	-
	100%	47.3%	0%	52.7%	100%	100%	0%	74.5%

Table S6. Genotyping Accuracy for Heterozygous Data, Minimum Flank Length 20. Results for four typical simulated read sets generated from the reference genome and mapped back to the reference TRs. Two sets of hg19 chromosomes were used to generate the reads. One set was unmodified, the other set was modified by changing the TR arrays to those in the 1118 modified ref-TRs. Simulated reads were drawn equally from the modified and unmodified chromosomes and mapped back to the unmodified reference set. Only those references with at least two spanning reads from both chromosomes (the minimum required to detect two alleles) were considered when determining sensitivity. Sensitivity of heterozygous VNTR calling is the percentage in column 3. Specificity of heterozygous VNTR calling is the percentage in column 7 and is essentially 100% in all cases. Given the large negative set size, an important measure is positive predictive value (PPV) in column 9. †Because the individual Illumina read simulation sets did not produce enough ref-TRs with at least two spanning reads in each chromosome set, the six sets (three exact, three error) were combined into two sets (one exact, one error) for this table.

Read Set	Read Mapping		Reference TR Mapping		Genotype Calling								
					Unmodified TR		Homozygous VNTR			Heterozygous VNTR†			
	Sen	Spec	Sen	Spec	Sen	Spec	Sens	Spec	PPV	Sens	Spec	PPV	
454 Exact (avg. 261 nt)	97.5%	99.6%	96.9%	99.2%	97.7%	100%*	95.8%	100%*	96.3%	84.2%	100%*	91.6%	
454 Errors (avg. 261 nt)	90.1%	99.5%	94.7%	99%	93.7%	99.9%	91.9%	100%*	91.8%	76.6%	100%*	85.8%	
Illumina Exact (100 nt)	94.5%	99.5%	96.8%	99.6%	95.0%	100%*	93.6%	100%*	98.1%	83.1%	100%*	86.5%	
Illumina Errors (100 nt)	70.4%	97.7%	78.1%	97.4%	67.6%	100%*	64.8%	100%*	94.2%	47.3%	100%*	74.5%	

Table S7. VNTRseek Accuracy, Minimum Flank Length 20. (Same as Table 1 in the paper.) Average accuracy measures for twelve simulated read sets, three each for two technologies (454 and Illumina) generated from the reference genome (Exact) and three each obtained by introducing errors into exact reads (Errors). Read Mapping is the accuracy of assigning reads to the correct reference TRs. Reference TR Mapping is the accuracy with which reference TRs were assigned reads. Genotype Calling is the accuracy of calling unmodified reference TRs and homozygous VNTRs in a modified reference set where 1118 randomly selected reference TRs (approximately 0.5% of the total) were modified by adding or subtracting one or two pattern copies, and the accuracy of calling heterozygous VNTRs where the unmodified reference set was used and reads were selected equally from two chromosome sets, one exact and one modified to match the modified references. PPV is positive predictive value, the fraction of called VNTRs that were correct. Typical data is shown in Supplementary Tables S2 - S6. *Specificity for unmodified TR calling and VNTR calling is slightly less than 100%. †Heterozygous VNTR values for Illumina reads obtained by combining three data sets into one in order to obtain enough ref-TR loci spanned by at least two reads in both the modified and unmodified chromosome sets.

Randomly Generated Reads				
Read Set	Reference TR Spanning		Other TR Spanning	
	Generated	Correctly Mapped	Generated	Incorrectly Mapped
454 Exact	955,880	932,774	1,844,506	5,823
	100%	97.6%	100%	0.3%
454 Errors	955,880	856,668	1,933,265	5,560
	100%	89.6%	100%	0.3%
Illumina Exact	449,121	423,197	1,016,789	3,711
	100%	94.2%	100%	0.4%
Illumina Errors	449,349	322,215	1,000,646	12,622
	100%	71.7%	100%	1.3%

Table S8. Read Mapping Accuracy, Minimum Flank Length 10. Results for four typical simulated read sets generated from the reference genome and mapped back to the reference TRs. Two sets are 454 type reads, two are Illumina type reads. Reads in the Exact sets exactly match the reference while reads in the Errors sets contain simulated sequencing errors. Reference TR Spanning reads are those that spanned the locus of a reference TR including at least ten nucleotides of flanking sequence on each side. Correctly mapped means the read was mapped to the correct reference TR. Other TR Spanning reads are those that contained a spanned TR, but not a reference TR. Incorrectly mapped in this group means the read was mapped to a reference TR when it should not have been mapped to anything. Sensitivity is the percentage in column 3. Specificity is $1-$the percentage in column 5. The 454 reads in all the simulations had an average length of 261 nt. Illumina reads in all the simulations had a length of 100 nt.

Reference TR Spanning Reads Not Correctly Mapped				
Read Set	All	Mapped Incorrectly	Failed TRF	Other
454 Exact	23,106	438	654	22,014
454 Errors	99,212	641	36,052	62,519
Illumina Exact	25,924	169	967	24,788
Illumina Errors	127,134	305	90,952	35,877

Table S9. Fate of Reference TR Spanning Reads Not Mapped Correctly, Minimum Flank Length 10. Results for four typical simulated read sets generated from the reference genome and mapped back to the reference TRs. A very small number of reads mapped to the wrong reference. In the reads with errors, reads were commonly discarded because the errors affected detection of the repeat by TRF. This was especially true in the shorter Illumina type reads, because the spanned TRs contained fewer copies and were more easily disrupted. Other reasons for discarding a read were a) ties – the read-TR mapped to more than one reference with equal score, b) multiple loci – different read-TRs in the same read mapped to two references that were not close enough together to be spanned by the read or mapped to three or more references no matter their spacing, c) the read was classified as a PCR duplicate, and d) TR profile or flank scores failed to meet the thresholds.

Reference TRs							
Read Set	With Spanning Reads	With Mapped Reads			Without Spanning Reads	With Mapped Reads	
		Only Correct	Other	None		None	Any
454 Exact	212,562	206,480	1,801	4,281	17,744	17,494	250
	100%	97.1%	0.8%	2.0%	100%	98.6%	1.4%
454 Errors	212,562	202,179	2,279	8,104	17,744	17,469	275
	100%	95.1%	1.1%	3.8%	100%	98.5%	1.5%
Illumina Exact	161,985	155,952	1,555	4,478	68,321	67,897	424
	100%	96.3%	1.0%	2.8%	100%	99.4%	0.6%
Illumina Errors	162,357	135,538	9,805	17,014	67,949	66,334	1,615
	100%	83.5%	6.0%	10.5%	100%	97.6%	2.4%

Table S10. Reference Mapping Accuracy, Minimum Flank Length 10. Results for four typical simulated read sets generated from the reference genome and mapped back to the reference TRs. Only those references with at least one spanning read were considered (92% of the references in the 454 reads and 70% of the references in the Illumina reads, both approximately as expected from Table S1.). Sensitivity is the percentage shown in column 3. Specificity is the percentage shown in column 7. Lower sensitivity in the Illumina reads with errors was primarily due to discarding reads in which TRF did not detect the repeat.

Generated Homozygous VNTRs					Other Reference TRs							
Read Set	2-span	Called		Not	2-span	Called		Other	Called		VNTR
		Correct	Incorrect	Detected		Correct	VNTR		No	VNTR	PPV
454 Exact	946	904	0	42	196,806	192,408	55	32,554	32,521	33	–
	100%	95.6%	0.0%	4.4%	100%	97.8%	0.0%	100%	99.9%	0.1%	91.1%
454 Errors	946	873	1	72	196,806	184,788	55	32,554	32,522	32	–
	100%	92.3%	0.1%	7.6%	100%	93.9%	0.0%	100%	99.9%	0.1%	90.8%
Illumina Exact	602	575	0	27	122,148	117,592	18	107,556	107,524	32	–
	100%	95.5%	0.0%	4.5%	100%	96.3%	0.0%	100%	100.0%	0.0%	92.0%
Illumina Errors	574	441	1	132	122,052	94,343	10	107,680	107,649	31	–
	100%	76.8%	0.2%	23.0%	100%	77.3%	0.0%	100%	100.0%	0.0%	91.3%

Table S11. Genotyping Accuracy for Homozygous Data, Minimum Flank Length 10. Results for four typical simulated read sets generated from the reference genome and mapped back to the reference TRs. 1118 randomly selected reference TRs (approximately 0.5% of the total) were modified by adding or subtracting one or two pattern copies. Only those references with at least two spanning reads were considered (the minimum required to call an allele) when determining genotype calling sensitivity. Specificity of VNTR calling is the ratio of all other reference TRs not called as VNTRs (230306 - column 2 - column 8 - column 11) to all other reference TRs (230306 - column 2) and is essentially 100% in all cases. Given the large negative set size, an important measure is positive predictive value (PPV), the ratio of true VNTR calls to all VNTR calls (column 12). In the 454 read set with errors, 9.2% of the VNTR calls were incorrect (approximately 1 out of 11). When subdivided, this corresponds to approximately 1 in 36 (24/868) incorrect calls for singletons and 1 in 14 (64/93) incorrect calls for indistinguishables. In the Illumina read set with errors, 8.5% of the VNTR calls were incorrect (approximately 1 out of 12). When subdivided, this corresponds to approximately 1 in 14 (33/468) incorrect calls for singletons and 1 in 2 (8/15) incorrect calls for indistinguishables. Sensitivity of unmodified TR calling is the percentage in column 7. Specificity of unmodified TR calling is the percentage in column 10.

Generated Heterozygous TRs					Other Reference TRs			
Read	2-span	Called Heterozygous				Called Heterozygous		
Set	both alleles	Correct	Incorrect	No	All	No	Yes	PPV
454 Exact	359	294	1	64	229,947	229,892	55	-
	100%	81.9%	0.3%	17.8%	100%	100%	0%	84.2%
454 Errors	359	267	0	92	229,947	229,894	53	-
	100%	74.4%	0%	25.6%	100%	100%	0%	83.4%
†Illumina Exact	330	265	0	65	229,976	229,870	106	-
	100%	80.3%	0%	19.7%	100%	100%	0%	71.4%
†Illumina Errors	330	213	0	117	229,976	229,889	87	-
	100%	64.5%	0%	35.5%	100%	100%	0%	71.0%

Table S12. Genotyping Accuracy for Heterozygous Data, Minimum Flank Length 10. Results for four typical simulated read sets generated from the reference genome and mapped back to the reference TRs. Two sets of hg19 chromosomes were used to generate the reads. One set was unmodified, the other set was modified by changing the TR arrays to those in the 1118 modified ref-TRs. Simulated reads were drawn equally from the modified and unmodified chromosomes and mapped back to the unmodified reference set. Only those references with at least two spanning reads from both chromosomes (the minimum required to detect two alleles) were considered when determining sensitivity. Sensitivity of heterozygous VNTR calling is the percentage in column 3. Specificity of heterozygous VNTR calling is the percentage in column 7 and is essentially 100% in all cases. Given the large negative set size, an important measure is positive predictive value (PPV) in column 9. †Because the individual Illumina read simulation sets did not produce enough ref-TRs with at least two spanning reads in each chromosome set, the six sets (three exact, three error) were combined into two sets (one exact, one error) for this table.

Read Set	Read Mapping		Reference TR Mapping		Genotype Calling								
					Unmodified TR		Homozygous VNTR			†Heterozygous VNTR		
	Sen	Spec	Sen	Spec	Sen	Spec	Sens	Spec	PPV	Sen	Spec	PPV
454 Exact	97.5%	99.7%	97.1%	98.7%	97.8%	99.9%	95.5%	100%*	91.6%	82.4%	100%*	84.0%
454 Exact	89.5%	99.7%	95.1%	98.6%	93.9%	99.9%	91.8%	100%*	90.6%	74.3%	100%*	83.6%
Illumina Exact	94.2%	99.6%	96.3%	99.4%	96.3%	100%*	94.7%	100%*	92.1%	80.3%	100%*	71.4%
Illumina Errors	71.6%	98.7%	83.5%	97.6%	77.3%	100%*	74.5%	100%*	93.3%	64.5%	100%*	71.0%

Table S13. VNTRseek Accuracy, Minimum Flank Length 10. Average accuracy measures for twelve simulated read sets, three each for two technologies (454 and Illumina) generated from the reference genome (Exact) and three each obtained by introducing errors into exact reads (Errors). Read Mapping is the accuracy of assigning reads to the correct reference TRs. Reference TR Mapping is the accuracy with which reference TRs were assigned reads. Genotype Calling is the accuracy of calling unmodified reference TRs and homozygous VNTRs in a modified reference set where 1118 randomly selected reference TRs (approximately 0.5% of the total) were modified by adding or subtracting one or two pattern copies, and the accuracy of calling heterozygous VNTRs where the unmodified reference set was used and reads were selected equally from two chromosome sets, one exact and one modified to match the modified references. PPV is positive predictive value, the fraction of called VNTRs that were correct. Typical data is shown in Supplementary Tables and S8 - S12. *Specificity for unmodified TR calling and VNTR calling is slightly less than 100%. †Heterozygous VNTR values for Illumina reads obtained by combining three data sets into one in order to obtain enough ref-TR loci spanned by at least two reads in both the modified and unmodified chromosome sets.

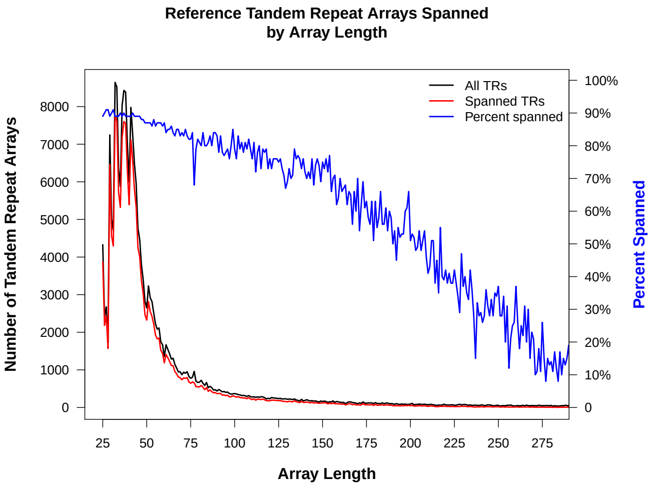

Figure S2. Reference TRs spanned by at least one read, Khoisan genome. "All TRs" is the number of reference TR loci at each array length (combined length of all copies in the TR). "Spanned TRs" have at least one read mapped by VNTRseek analysis. "Percent spanned" is the ratio of spanned loci to all loci. Percent spanned for the Khoisan genome is significantly higher than for the Watson genome (compare Figure 2 from paper) due to the longer read length and higher coverage.

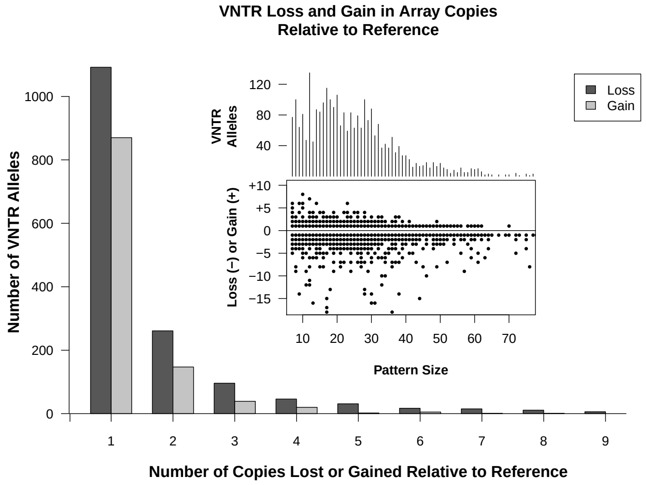

Figure S3. Khoisan VNTRs. Large graph: Distribution of copy loss or gain relative to the reference. **Inset top:** Distribution of VNTRs by pattern size. **Inset bottom:** Number of copies gained or lost, by pattern size. Note that frequency for each gain or loss is not shown, only occurrence. Data is for 2,698 alleles from 2,572 reference TRs called as VNTRs. (Omitted from large graph are 33 VNTRs with loss/gain greater than 9. Omitted from offset bottom are ten VNTRs with loss greater than 18 copies and one with gain equal to 16 copies. Omitted from both insets are 20 VNTRs with pattern size ≥ 77 nt.)

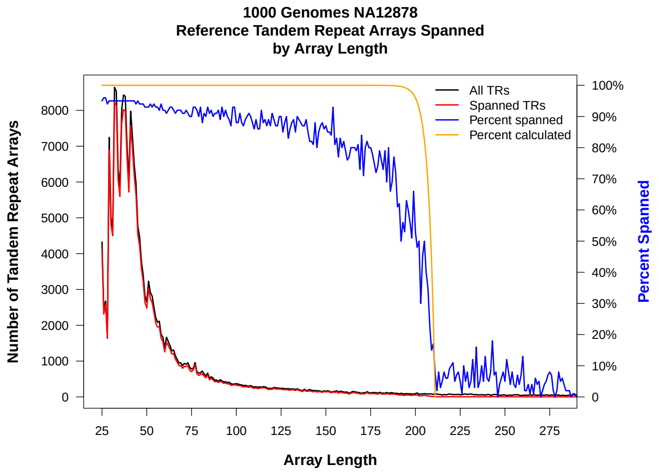

Figure S4. 1000 Genomes NA12878, reference TRs spanned by at least one read, expected versus observed. "All TRs" is the number of reference TR loci at each array length (combined length of all copies in the TR). "Spanned TRs" have at least one read mapped by VNTRseek analysis. "Percent spanned" is the ratio of spanned loci to all loci. "Percent calculated" is an expected value for percent spanned derived using a probability formula for autosomal TR arrays, a fixed read length equal to 250nt, and 816,000,000 reads, the number in the data set for this genome. Coverage was approximately 68. The steep drop-off at 185-210 nt is due to the fixed read length. The small percentage spanning above 210 nt (jagged blue line to the right of the vertical part of the orange line) is due to reads mapping to 304 long arrays in the reference that appeared shorter in the reads.

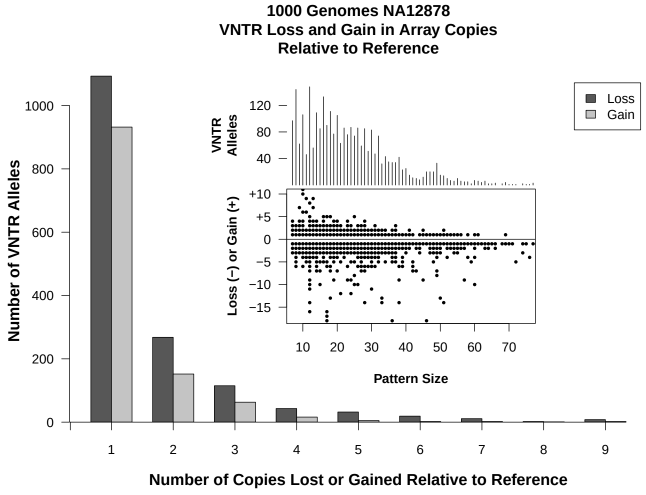

Figure S5. 1000 Genomes NA12878 VNTRs. Large graph: Distribution of copy loss or gain relative to the reference. **Inset top:** Distribution of VNTRs by pattern size. **Inset bottom:** Number of copies gained or lost, by pattern size. Note that frequency for each gain or loss is not shown, only occurrence. Data is for 2,812 alleles from 2,659 reference TRs called as VNTRs. (Omitted from large graph are 34 VNTRs with loss/gain greater than 9. Omitted from inset bottom are seven VNTRs with loss greater than 18 copies and one with gain equal to 18 copies. Omitted from both insets are 17 VNTRs with pattern size ≥ 77 nt.)

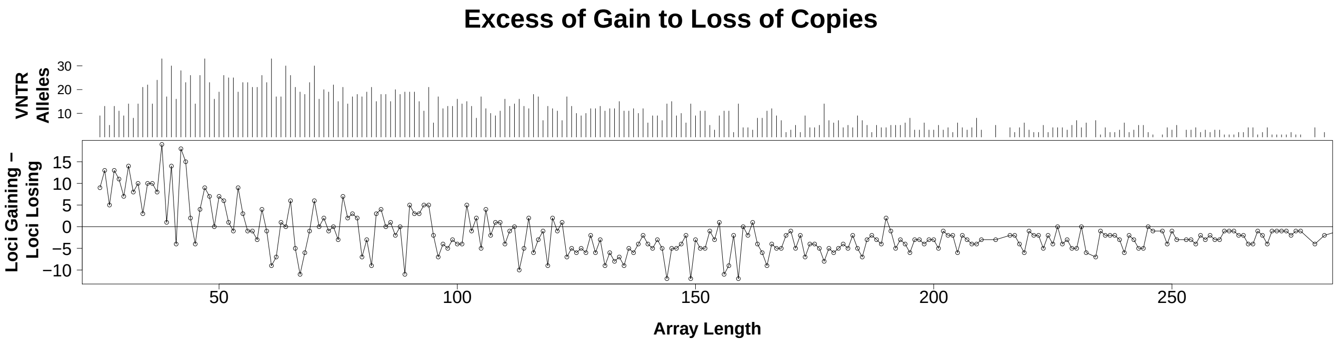

Figure S6. Khoisan VNTRs. Top: Distribution of VNTRs by array length in the reference. Bottom: Difference between number of loci that show copy gain and number that show copy loss, relative to the reference. Below about 50 nt, VNTRs show an excess of gain over loss. Above 50 nt, there is a gradual shift from neutral to loss, as expected because of the limited read size. Contrast with Watson data, Figure 4 in the paper, which shows an abrupt change from gain to loss at 50 nt. (Omitted from the graph are 118 VNTRs with reference array length longer than 282 nt.)

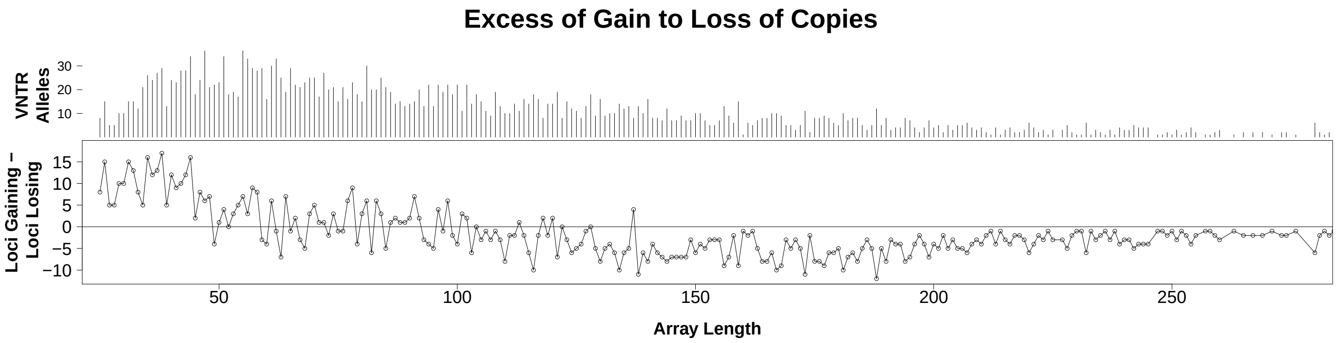

Figure S7. 1000 Genomes NA12878 VNTRs. Top: Distribution of VNTRs by array length in the reference. Bottom: Difference between number of loci that show copy gain and number that show copy loss, relative to the reference. Trend from gain to loss with increasing array length is similar to that for the Khoisan genome. (Omitted from the graph are 76 VNTRs with reference array length longer than 283 nt.)

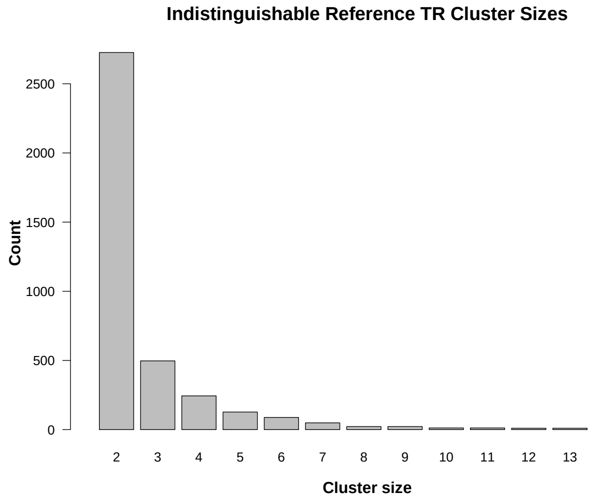

Figure S8. Indistinguishable Reference Cluster Sizes. 13,941 ref-TRs were classified as indistinguishable. Over half occurred in clusters of four or fewer references. 26% occured in clusters larger than the maximum size shown here (13).

A. Mapping

	Total	After Filters	Mapped	%
Ref-TRs	1,188,939	230,306	189,627	82
Read-TRs	87,198,666	59,601,310	1,352,594	2
Reads	83,331,227	15,009,889	1,318,000	8

B. Mapped Reference Results

Number of Reads Mapped		At Least One Allele Supported	By Reference Category	
$\geq$ One	$\geq$ Two		Singleton	Indist.
189,627	174,907	174,767	184,453	5,174
100%	92%	92%	97%	3%

C. VNTR Results

	Alleles Supported				By Reference Category	
	One	Two or More				
	⋆	•	•	•		
Total	Diff	Same/Diff	Diff/Diff	Multi	Singleton	Indist.
2,572	1,626	828	112	6	2,465	107
100%	63%	32%	4%	0%	96%	4%

⋆ Inferred VNTR • Observed VNTR

Table S14. Khoisan VNTRseek Results. A. Input data and data after filtering the reference set and the read set; B. Counts and percentages of mapped references that were assigned at least one read, at least two reads, had at least one allele supported, and were either singleton or indistinguishable. C. Counts and percentages of total VNTRs, number of alleles supported, and reference category. Six VNTRs had three alleles (Multi) which is not expected. Two of these are indistinguishables and the other four are likely indistinguishables whose other family members were filtered from the reference set.

A. Mapping

	Total	After Filters	Mapped	%
Ref-TRs	1,188,939	230,306	202,469	87
Read-TRs	163,283,393	60,350,672	3,593,180	6
Reads	816,360,354	33,279,934	3,537,602	11

B. Mapped Reference Results

Number of Reads Mapped		At Least One Allele Supported	By Reference Category	
$\geq$ One	$\geq$ Two		Singleton	Indist.
202,469	198,697	198,617	195,276	7,193
100%	98%	98%	96%	4%

C. VNTR Results

	Alleles Supported				By Reference Category	
	One	Two or More				
	⋆	•	•	•		
Total	Diff	Same/Diff	Diff/Diff	Multi	Singleton	Indistinguishable
2,659	1,403	1,119	122	15	2,442	217
100%	52%	42%	4%	0%	91%	8%

⋆ Inferred VNTR • Observed VNTR

Table S15. NA12878 VNTRseek Results. A. Input data and data after filtering the reference set and the read set; B. Counts and percentages of mapped references that were assigned at least one read, at least two reads, had at least one allele supported, and were either singleton or indistinguishable. C. Counts and percentages of total VNTRs, number of alleles supported, and reference category. 14 VNTRs had three alleles supported and 1 had four alleles supported (Multi) which is not expected. Six of these are indistinguishables and the other nine are likely indistinguishables whose other family members were filtered from the reference set.
